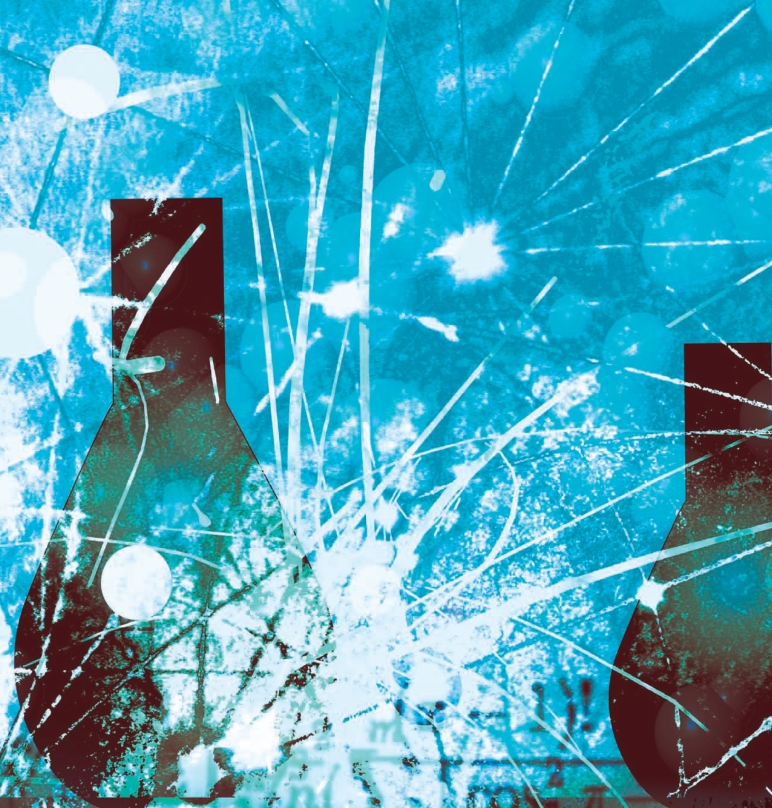# Chemical Reaction: The U.S. Response to REACH

**DOI:** 10.1289/ehp.116-a124

**Published:** 2008-03

**Authors:** Harvey Black

Last summer ushered in a new era in the regulation of chemicals. On 1 June 2007, REACH (Registration, Evaluation, Authorisation and Restriction of Chemicals), the expansive scheme by the European Union (EU) to regulate chemicals used in commerce and consumer products, took effect. REACH applies to chemicals manufactured or marketed in Europe, and its regulations affect companies exporting chemicals to Europe as well as those located there. REACH puts the burden on chemical companies to provide information on how the chemicals they make affect human health and the environment. REACH has two parts: the collection and sharing of data throughout supply chains, and the authorization of chemicals of higher concern to human and environmental health.

In an initiative that is set to be phased in over the next 11 years, REACH will require the registration of chemicals produced or marketed in the EU in quantities of 1 metric ton or greater per year. Chemicals imported or produced in amounts of 1,000 metric tons or more are to be registered by November 2010, whereas those at amounts of 1 metric ton or more are to be registered by May 2018.

“The basic philosophy of REACH is that the [chemical] industry is managing the risk, and what REACH does is require the industry to put on paper the knowledge about the chemicals they put on the market, and describe how they are dealing with any possible risk which might be in them,” says Joachim Kreysa, director for cooperation at the European Chemicals Agency (ECHA), which administers REACH.

Chemical companies have from 1 June 2008 until 30 November 2008 to pre-register so-called phase-in substances—ones that are already marketed in the EU, or that have been imported or made in the EU in the past 15 years even if not sold there—by providing ECHA with such basic information as the name of the chemical and the importer. “It is important that companies [outside Europe] begin to consider the possible impact of REACH on their business now,” says Malachy Hargadon, environmental counselor with the European Commission, the executive branch of the 27-nation EU. These companies should be examining their stock of chemicals and the requirements of REACH, he adds. Such chemicals are in a database called EINECS, or European Inventory of Existing Chemical Substances.

## Compliance Requirements

REACH requires safety and exposure data, including new testing in some cases, on an estimated 30,000 chemicals that will be sold in Europe. The extent of the data required increases with increasing production volume. Compiling that information is likely to be a mammoth task. “There’s never been a data set compiled for this many chemicals like this in history,” says Spencer Williams, a toxicologist at ChemRisk, a Houston, Texas–based consulting firm that is helping U.S. chemical companies understand what REACH compliance involves.

REACH also requires chemical manufacturers to submit a chemical safety report, or CSR, for the approximately one-third of chemicals that are imported or produced in quantities greater than 10 metric tons per year. The goal of the CSR is to understand the exposure scenario for each use and demonstrate that these risks can be adequately controlled. The CSR includes a human health and environmental hazard assessment of a chemical and a determination of how persistent and bioaccumulative it is. The CSR must also include information on how the chemical is used by downstream users—industries that use the chemical in the products they make—and the risks for different exposure scenarios. By the same token, REACH requires the downstream user to give the chemical manufacturer enough information to allow it to assess the substance’s safety in the context of each use, or the user may perform its own CSR. The result, says Charles Bartish, director of product safety and regulatory compliance at gas manufacturer Air Products and Chemicals, Inc., can be a document of between 10 and 100 pages, which he says can be a significant burden. REACH affects several hundred chemicals that Air Products supplies to the industries the company serves.

## Different Effects for Different Companies

Williams says the impact of REACH spans a spectrum, differentially affecting small and large companies and requiring a significant commitment of skilled professionals. Small companies may work hard to comply with REACH information requirements, “but they’re going to be behind the eight ball in comparison to their big competitors. Dow [Chemical Company] has a number of people in-house, at least eighteen that I’m aware of, who are hired to work on REACH,” he says. Smaller companies, he says, are likely to struggle because they simply lack the staff.

Williams says there are many tasks involved in compliance, such as performing cost–benefit analyses for chemicals (to determine whether it is worth the cost to register chemicals with REACH), understanding the supply chain (who gets what chemicals, how are they used, etc.), gathering information for the CSR, arranging for studies on exposures to and hazards of chemicals, and gathering information from existing studies on exposure and hazard.

The U.S. Department of Commerce is reaching out to help small and medium-sized companies, says Rosemary Gallant, a Brussels-based commercial attaché of the department. “Our objective is to make sure small and medium-sized exporters can stay in the market. Our goal is to help them understand what they need to know about REACH, and make them aware of the time frame for REACH,” she says. She notes that some smaller U.S. exporters feel as though they are being picked on by having to comply with REACH requirements and are unaware that REACH also applies to European chemical companies as well.

Williams notes that REACH may mean certain chemicals will no longer be used in Europe. For example, he is familiar with one company that is considering whether it is even worthwhile to pre-register certain chemicals that it sells in Europe; the costs in terms of manpower and fees paid to ECHA may simply not be worthwhile relative to the amount of money the chemicals may earn.

But Europe is a major market for chemical companies, says Geoffrey Bock, regional sales manager for TÜVRheinland, a firm that offers compliance help to companies affected by REACH. According to the European Commission, Europe is a €10 billion (US$14 billion) economy representing more than 490 million consumers. When that is factored in, companies realize it is worth the time and effort to comply with REACH. “You have to remain competitive,” Boc says.

Bartish notes that in spite of the amount of information his company will have to supply to comply with REACH, it is not considering dropping any of the chemicals it makes from European markets.

Some companies have expressed concern about the substance information exchange forum (SIEF) process that grows out of pre-registering chemicals. ECHA will group together companies who have pre-registered similar substances. Those companies, many of which are competitors, will have to work together to provide much of the detailed information required to actually register a substance.

Michael Walls, managing director of health, products, and science policy at the American Chemistry Council, claims this process will force companies to share confidential business information. Furthermore, says U.S. Environmental Protection Agency (EPA) policy advisor Christopher Blunck, that agency would be able to use publicly available data developed by chemical companies in its regulatory efforts. The EPA could also gain access to confidential business information submitted under REACH, says Richard Denison, a senior scientist with the Environmental Defense Fund. And unlike the Toxic Substances Control Act, the U.S. counterpart to REACH, the latter allows the EU to share such information with other national governments.

But Kreysa disputes that concern. “The data exchange is one hundred percent controlled by the companies themselves. We are not forcing them to exchange anything that is confidential,” he says. If the data exchange cannot be done without providing confidential business information, he says, companies can opt out provided they can justify their reasons to ECHA.

Moreover, such groupings of chemical companies are nothing new, says Denison. The chemical industry has fostered these groups to provide the EPA and the Organisation for Economic Co-operation and Development with testing and related data under their programs that call for data on high-production-volume chemicals (see “GAO Sounds Off on Chemical Regulation,” *EHP* 113:A828–A830 [2005]). “That is essentially what the SIEFs are,” Denison says. “They are an effort to try to ensure that we don’t have duplicative or unnecessary testing or requirements imposed.”

## Focus on Toxicity

REACH focuses regulatory attention on so-called substances of very high concern, which include those that are carcinogenic, mutagenic, persistent, bioaccumulative, or toxic to reproduction. They can also include other substances for which there is “scientific evidence of serious effects to human health and the environment,” says Kreya. “This will be determined on a case-by-case basis.” He adds that substances of very high concern cannot be marketed without specific authorization from ECHA.

Walls finds REACH’s focus on these substances disturbing and somewhat misplaced, arguing that the possibility of removing chemicals from the market simply because of their “hazard characteristics” is unnecessary. The issue of importance, he says, is exposure, not hazard in and of itself. “We use hazardous chemicals everyday,” he says.

But Denison argues that the distinction between exposure and risk is not as clear as Walls would have it. “Our ability to predict exposure is notoriously bad,” he says. He cites brominated flame retardants as an example of a hazardous chemical to which exposure was not predicted, yet the chemicals are routinely found in humans and wildlife. A June 2007 study in *EHP* reported that neonatal exposure to the flame retardant BDE-47 impaired the neurodevelopment of mice.

Denison also argues that, even if a chemical company is able to show there is low exposure to a chemical in its production facility, that chemical may be sold to customers who may use it in ways that the maker has no information about—a contingency REACH attempts to address with its CSR requirement.

Walls acknowledges that the information sharing promoted by REACH can be valuable, saying, “The responsibility to safely manage chemicals is not just with the original producer, it’s also with the downstream user.” He adds, “REACH requires far more detailed exposure information from downstream users. The benefit of what REACH will do is force us to understand nearly all the end-use applications and see if there are any applications that cause us concern.”

Kreysa notes that ECHA can grant authorization to market a hazardous chemical if the company shows it can successfully manage the risk by keeping exposure to a minimum. But such authorization does have limits. The most hazardous substances of very high concern can be authorized only if the applicant shows that the chemical’s benefits outweigh its risks and that there are no viable alternatives.

As for alternative chemicals, ECHA has established committees to perform socioeconomic analyses of such replacements and to consider risk management measures. These committees “will deliver an opinion if these measures are going to reduce the risk sufficiently,” Kreysa says. Furthermore, permission to use such hazardous chemicals is allowed only for specified time periods. “The intention is that substances of very high concern are to be replaced. But there is no automatic deadline [for replacement],” he says.

## Costs and Benefits of Compliance

According to an EU fee schedule issued late in 2007, standard costs for registering chemicals are determined on a sliding scale, with €31,000 (about US$45,000) currently the cost of a chemical in the 1,000-metric-ton-or-more group; the cost drops to €1,600 (US$2,300) for a substance in the 1- to 10-metric-ton group. These fees are reduced for small and medium companies. The cost of applications for authorization of substances of very high concern is expected to be around €50,000 (US$72,000) for each use of the chemical. The cost of preparing the chemical dossier will be several times that.

Walls says these costs are a major concern for companies. “What you’re talking about is a significant financial incentive to eliminate chemicals,” he says. Furthermore, he argues the financial cost will interfere with companies investing in innovation and developing new products.

This is completely untrue, counters Denison: REACH actually decreases requirements for registering new chemicals, compared with prior EU rules, while significantly increasing requirements for existing chemicals. Denison says a major motivation of REACH was to level the playing field between new and existing chemicals—precisely to remove the earlier disincentive to introduce new chemicals.

Participants at a workshop on REACH impact assessment, which was held 25–27 October 2004 and organized by the Dutch presidency for the EU in The Hague, acknowledged concerns about the legislation’s impact on innovation. Innovation may be affected, concluded the workshop summary report, *The Impact of Reach*, “because available resources for research and development might be used for the implementation of REACH,” particularly by small, medium, and exporting companies. But the workshop concluded that is only a short-range impact. Moreover, the savings in health were predicted to far outweigh cost, and “in the longer perspective, the requirements of REACH may stimulate the development of less harmful substances as substitutes for restricted ones.”

Joel Tickner, a director at the Lowell Center for Sustainable Production at the University of Massachusetts Lowell, foresees downstream users of chemicals forcing innovation. “REACH will give users of chemicals—companies that don’t really need [a specific] chemical but its functionality—the opportunity to push markets toward safer chemistry,” he says. And he views the argument that REACH may stifle innovation as specious: “If you look at the chemical universe right now, of the chemicals on the market today, ninety-nine percent by volume were on the market in 1979.”

In fact, Tickner argues, regulation can actually drive innovation. He says there is a clear interest of downstream users of chemicals who want the functionality of the chemicals but not their toxicity. Companies in sectors such as health care, footwear, electronics, and cleaning chemicals have already started to demand these products from suppliers. “REACH will provide important information to help distinguish safer and less safe chemicals,” Tickner says. He points to the Green Chemistry and Commerce Council, a network of companies dedicated to green chemistry and sustainable design, which has been working extensively to effectively promote these concepts and whose participants realize the critical importance of REACH type data.

As a case in point, the EU’s July 2006 ban on lead solder in electronics helped spur the development of alternatives that don’t use lead, says Raymond Lizotte, a product environmental compliance engineer at American Power Conversion Corporation in Massachusetts. Fine-tuning alternatives can take additional time. Still, Lizotte noted in the December 2007 report *CleanTech: An Agenda for a Healthy Economy* that his company had been working to develop lead-free solder since the early 1990s, with little to show for it until the ban came about: “While our preceding efforts allowed us to meet [the EU] deadlines successfully, it’s hard to discount the role that the regulatory requirement played in finally bringing lead-free products to market.”

Williams notes American chemical companies “are not crazy about REACH,” but he says they also recognize that it is a set of regulations they have to live with if they wish to do business in Europe. “That’s really been a development in the last three or four months. A lot of them are getting over their shock and figuring out what needs to be done,” he says. Tickner says the time spent by the U.S. government and the chemical industry arguing against REACH has put U.S. companies several years behind their European colleagues in preparing for compliance, which could put U.S. companies at a disadvantage.

At least one major player sees REACH as potentially beneficial. Dow Chemical Company stated on its “Dow and REACH” website (http://www.dow.com/reach/) that the new policy “represents a significant opportunity for chemicals manufacturers, their suppliers, and customers to work together to protect the environment and preserve the future of the chemicals industry in Europe.” Dow spokesman Mark Walton noted that the information required by REACH would help protect health and the environment by “helping to identify and alleviate situations where exposure to chemicals may be at levels that should be reduced.” The overall impact of REACH, according to Walton, would be a “more favorable and sustainable business climate for Dow and the chemical industry.”

Kreysa expects the information required by REACH to help ensure better public and environmental health. “We will know a lot more about the chemicals that are on the market and the potential problems they might pose,” he says. Because the firms that are the final users of the chemical will know much more, they will be able to better manage risk. “This should reduce the exposure of humans and the environment to chemicals that have negative effects,” he says.

Denison says the information provided by REACH should begin to help overcome the difficulty in linking specific health problems with exposures to specific chemicals or mixtures of chemicals. He says it’s important to increase understanding of the hazards posed by certain chemicals, and to take steps to avoid exposing people to such chemicals. “That’s what I think REACH is trying to do,” he says.

## A Spreading Influence

REACH’s influence is not stopping with chemical companies but is extending into state governments in the United States. For instance, Maine’s search for a more comprehensive way to regulate chemicals instead of a substance-by-substance and use-by-use approach was influenced by REACH, says Ginger Jordan-Hillier, environmental public health coordinator at the state’s Department of Environmental Protection. An executive order issued by Maine’s governor in February 2006 established a task force to come up with an overall policy requiring and offering incentives to develop safe chemicals in consumer products.

And in August 2007, in what could be thought of as a North American response to REACH, the United States, Canada, and Mexico signed an agreement in Montebello, Québec, to assess 9,000 chemicals produced or imported in volumes of 25,000 pounds or more. The countries are required to complete risk characterization on these chemcials by 2012. By 2020 the countries must have inventoried all chemicals currently used in commerce. The agreement is aimed at sharing information and coordinating risk management of the chemicals.

In California, REACH is “definitely being considered as a model” for chemical regulation, says Denison, who is a scientific advisor for the Green Chemistry Initiative, an effort by the California Department of Toxic Substances Control to promote green chemistry and identify toxic substances. “I think it can absolutely be said that the existence of REACH is influencing how California and no doubt other states are thinking about chemicals and chemicals policies,” says Denison.

REACH has, of course, just gone into effect. It is less than a year old, and its ambitious goals remain just that—goals. What remains uncertain, and may so remain for many years until REACH is fully implemented, is whether the promise of improved risk management of chemicals and improved environmental and public health will be realized.

## Figures and Tables

**Figure:**